# 10 Years of Environmental Change on the Slopes of Mount Kilimanjaro and Its Associated Shift in Malaria Vector Distributions

**DOI:** 10.3389/fpubh.2016.00281

**Published:** 2016-12-21

**Authors:** Manisha A. Kulkarni, Rachelle E. Desrochers, Debora C. Kajeguka, Robert Diotrephes Kaaya, Andrew Tomayer, Eliningaya J. Kweka, Natacha Protopopoff, Franklin W. Mosha

**Affiliations:** ^1^School of Epidemiology, Public Health and Preventive Medicine, University of Ottawa, Ottawa, ON, Canada; ^2^HealthBridge, Ottawa, ON, Canada; ^3^Kilimanjaro Christian Medical University College, Moshi, Tanzania; ^4^Tropical Pesticide Research Institute, Arusha, Tanzania; ^5^Catholic University of Health and Allied Sciences, Mwanza, Tanzania; ^6^London School of Hygiene and Tropical Medicine, London, UK

**Keywords:** *Anopheles arabiensis*, Tanzania, highland malaria, environmental change, ecological niche modeling, Maxent, geographic information systems, vector-borne diseases

## Abstract

**Introduction:**

Malaria prevalence has declined in the Kilimanjaro region of Tanzania over the past 10 years, particularly at lower altitudes. While this decline has been related to the scale-up of long-lasting insecticidal nets to achieve universal coverage targets, it has also been attributed to changes in environmental factors that are important for enabling and sustaining malaria transmission.

**Objectives:**

Herein, we apply spatial analytical approaches to investigate the impact of environmental and demographic changes, including changes in temperature, precipitation, land cover, and population density, on the range of the major malaria vector species *Anopheles arabiensis* in two districts of Tanzania, situated on the southern slope of Mount Kilimanjaro. These models are used to identify environmental changes that have occurred over a 10-year period and highlight the implications for malaria transmission in this highland region.

**Methods:**

Entomological data were collected from the Hai and Lower Moshi districts of Tanzania in 2001–2004 and 2014–2015. Vector occurrence data were applied alongside satellite remote sensing indices of climate and land cover, and gridded population data, to develop species distribution models for *An. arabiensis* for the 2004 and 2014 periods using maximum entropy. Models were compared to assess the relative contribution of different environmental and demographic factors to observed trends in vector species distribution in lowland and highland areas.

**Results:**

Changes in land cover were observed in addition to increased population densities, increased warm season temperature, and decreased wetness at low altitudes. The predicted area and extent of suitable habitat for *An. arabiensis* declined across the study area over the 10-year period, with notable contraction at lower altitudes, while species range in higher altitude zones expanded. Importantly, deforestation and warmer temperatures at higher altitudes may have created stable areas of suitable vector habitat in the highlands capable of sustaining malaria transmission.

**Conclusion:**

We show that environmental changes have had an important influence on the distribution of malaria vector species in a highland area of northern Tanzania. Highland areas may be at continued risk for sporadic malaria outbreaks despite the overall range contraction of principal vector species at lower altitudes, where malaria transmission remains at low intensity.

## Introduction

The global burden of malaria has declined considerably in the last two decades, with a 47% reduction in malaria mortality rates worldwide since 2000 and 54% reduction in the WHO Africa Region (1). While globally the decline has been attributed to economic development and public health interventions (2), local-scale changes in malaria risk have been attributed to changes in environmental factors that are important for enabling and sustaining malaria transmission (3–5). The local impacts of environmental change are particularly evident in the East African highlands, one of the most densely populated regions of Africa (6). These areas may be at heightened risk of malaria transmission due to multiple environmental, epidemiological, and socioeconomic factors that contribute to population vulnerability (7).

The impact of global climate change on malaria transmission has been widely debated (2, 8–11), although the dependence of malaria transmission on climatic variables, particularly temperature and rainfall, is well described (12). Rainfall principally influences the availability of surface water for mosquito breeding sites, although heavy rainfall may wash out breeding sites leading to fewer malaria cases, as was observed in Tanzania following the 1997–1998 El Nino event (13). Temperature exerts a key influence on vector and parasite development rates as well as vector survival and biting rates (14). Thus, while warmer temperatures and higher rainfall are often associated with increases in malaria incidence, the association is nuanced by ecological interactions (12). A recent multi-malaria model comparison study has provided evidence of climate change-driven contraction of the malaria transmission season over the Sahel and a southward shift of the malaria epidemic belt, while suggesting that future climate might become more suitable for malaria transmission in the tropical highland regions (15). It has been suggested that climate change will, without mitigation, result in an increase of the malaria burden in the densely populated highlands of Africa and South America (16).

In addition to climate change, anthropogenic environmental changes such as deforestation and urbanization, in addition to agricultural expansion and intensification, may have significant effects on mosquito habitat availability and hence malaria risk (17–19). These environmental changes have been shown to alter microclimatic conditions of the aquatic habitats that sustain *Anopheles* larval development and the indoor and outdoor habitats where adult mosquitoes rest, while further contributing to increases in vectorial capacity and heightened malaria transmission potential (20).

In Tanzania, a dramatic decline in malaria prevalence has occurred during the past decade, falling from 18 to 9% prevalence of parasitemia in children under five nationally between 2008 and 2012 (1). The observed decline was particularly notable in malaria endemic coastal areas, such as the Tanga region, which experienced a reduction in the prevalence of malaria parasitemia in lowland villages from 78 to 13% between 2003 and 2008 (21, 22). However, despite the overall trend in malaria decline, considerable variation in endemicity still exists in many areas, particularly in relation to altitude gradients (23–25). In the Kilimanjaro region of Tanzania, where the average prevalence of malaria parasitemia among children was less than 1% in 2011–2012 (26), community-based surveys and geostatistical models of national survey data have demonstrated marked altitudinal variations in malaria prevalence (23, 27, 28). In the early 2000s, community-based measures of malaria prevalence in districts surrounding Mount Kilimanjaro ranged from 6 to 13% in lowland populations (<800 m) and 0–3% in highland populations (800–1,800 m) (23, 27), while recent estimates in lowland and highland villages were <1 and 0%, respectively (D. Kajeguka, unpublished data). It has been noted that highland populations in this region have high biological susceptibility to malaria owing to their lack of previous exposure (29), highlighting the importance of identifying modifiable and non-modifiable factors that may contribute to malaria risk in highland populations, in order to mitigate public health impacts. Herein, we employ ecological niche modeling with satellite remote sensing data to investigate the changes in malaria vector distributions over a 10-year period in the Kilimanjaro region of Tanzania and identify the role of environmental and population changes, in order to understand the level and potential drivers of malaria risk in highland populations.

## Materials and Methods

### Study Site

The study area is located within the Hai and Moshi districts in the Kilimanjaro region of Tanzania, situated on the southwestern slope of Mount Kilimanjaro. This area has a population of approximately 850,000 (30) with villages and towns spanning an altitude range of approximately 600–1,800 m, including the major municipality of Moshi at approximately 900 m a.s.l. (population 184,000). The area receives between 900 and 1,200 mm of rainfall per year with two rainy seasons, the long rains that occur from March to May and short rainy season from November to December. *Anopheles arabiensis* has historically been recognized as the principal vector of *Plasmodium falciparum* malaria in the study area owing to its high seasonal densities, observed human biting tendency (human blood index ~50%), and sporozoite rate (~1–2%), with *A. funestus* s.l. playing a secondary role (31, 32). The Tanzania HIV and Malaria Indicator Survey estimated 1 and 0% malaria prevalence in children in the Kilimanjaro region in 2007–2008 and 2011–2012, respectively, although this does not reflect intraregional variation (26, 33). Household ownership of at least one insecticide-treated net (ITN) was 30% in 2007–2008 rising to 95% in 2011–2012; while only 1.8 and 0.3% of households reported having indoor residual spraying in the past 12 months in 2007–2008 and 2001–2012, respectively (26, 33). Malaria prevalence in this area has declined considerably: in 2001, it was classified at different altitude levels as moderately high or “mesohyperendemic” (750–1,000 m), moderate or “mesoendemic” (1,001–1,250 m), low moderate or “hypoendemic” (1,251–1,500 m), and non-endemic (>1,500 m) (27). Studies in the same area in 2015–2016 estimated <1% prevalence of malaria parasitemia in local populations (750–1,000 m) (D. Kajeguka, unpublished data).

**Figure 1:**
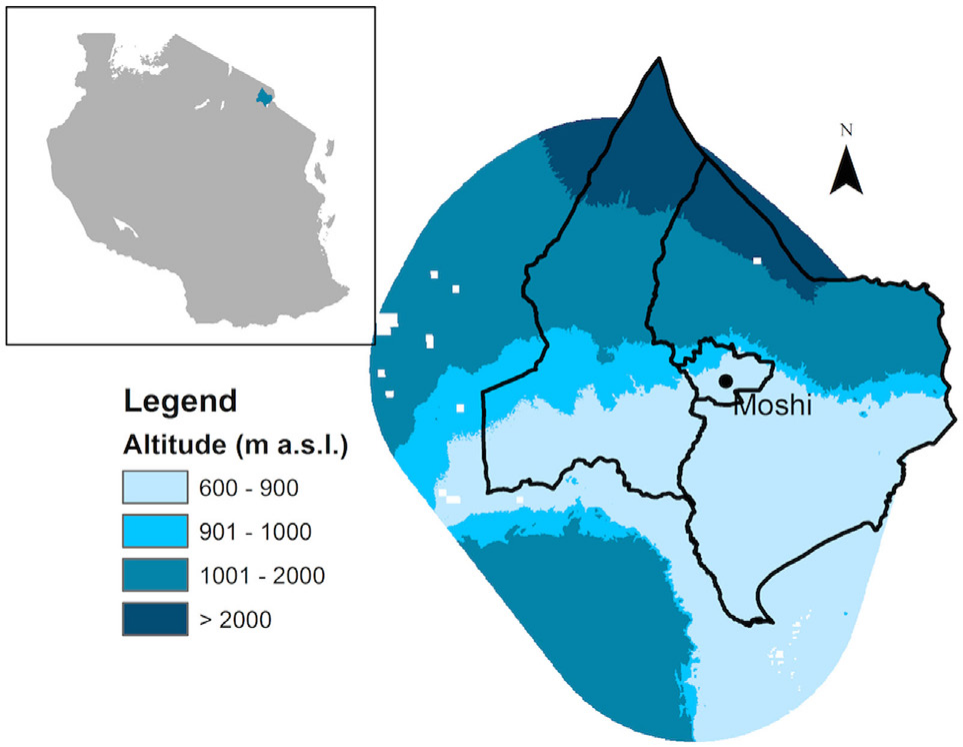
**Map of study area in the Hai and Moshi districts of Tanzania showing elevation zones**.

To assess changes in environmental factors and vector distributions over time, the study area was divided into three altitude zones: lowland (<900 m), mid-altitude (901–1,000 m), and highland (1,001–2,000 m) (Figure 1). Areas above 2,000 m are contained within Mount Kilimanjaro National Park where there is no human settlement.

### Mosquito Data

Historical occurrence records for *An. arabiensis* were obtained from prior studies conducted between 2001 and 2004 in the Hai and Moshi districts (24, 32). To measure the change in *An. arabiensis* distributions over a 10-year period, the two districts were revisited between July 2014 and August 2015 in the months following the short and long rainy seasons, and approximately 50 sites were sampled in each altitude zone. *Anopheles* mosquitoes were collected inside human dwellings using a CDC miniature light trap (Bioquip, Inc.) operated by battery from dusk to dawn (37 sites in 2001–2004 and 102 sites in 2014–2015). In 2014–2015, light trap collections were supplemented with Mosquito Magnet^®^ Liberty traps operated outdoors from mid-day to dusk in 54 sites to sample exophagic vectors. Collections in 2014–2015 were conducted in each site for two consecutive nights. All specimens were morphologically identified as *An. gambiae* s.l. (34) and were further identified to sibling species level by real-time polymerase chain reaction following the protocol described by Bass and others (35).

### Remote Sensing and Spatial Data

Satellite remote sensing and other spatial data were obtained for two time periods, 2004 and 2014–2015 (or the most recently available data), to investigate the changes in environmental factors and vector distributions across the study area. These years were selected to best reflect the periods of vector sampling and data availability.

For each time period, four environmental layers were obtained from NASA’s Moderate Resolution Imaging Spectroradiometer (MODIS), including land cover, vegetation indices [normalized differential vegetation index (NDVI); enhanced vegetation index (EVI)], and land surface temperature. The most recent land cover data available were for 2012, and this was taken as a proxy for land cover in 2014 as substantial changes are unlikely over a 3-year period. The land cover layers were available at a resolution of 500 m. Vegetation indices, such as NDVI and EVI, are highly correlated with precipitation (36, 37). Meteorological records from the Kilimanjaro International Airport weather station in 2002 and 2014 were used to identify the hottest/coldest and wettest/driest months for each year, defined as those with maximum/minimum mean values of temperature and rainfall, respectively. Mean monthly values were then calculated for temperature and vegetation indices to develop bioclimatic variables for model development: mean *T* of the hottest month (*T*_H_), mean *T* of the coldest month (*T*_C_), NDVI of the wettest month (NDVI_W_), NDVI of the driest month (NDVI_D_), EVI of the wettest month (EVI_W_), and EVI of the driest month (EVI_D_). Eight-day composites at 1 km resolution for land surface temperature and 16-day composites at 250 m for the vegetation indices were used to calculate the monthly means.

Elevation data were obtained from the Shuttle Radar Topography Mission at a resolution of 90 m, and human population density for 2004 and 2014 from the Oak Ridges National Laboratory Landscan database (http://web.ornl.gov/sci/landscan/), available at 1 km resolution. All data were reprojected to geographic WGS84 with a cell size of 0.0009 decimal degrees (approximately equivalent to 100 m) and clipped to the dimensions of the study area.

### Vector Niche Model Development

Maximum entropy (Maxent) software (38) was used to predict the distribution of *An. arabiensis* over the study area, following the approach described by Kulkarni et al. (24). Briefly, data were randomly partitioned for model evaluation, with 70% of the records used as training data to construct the models and the remaining 30% set aside for testing. The accuracy of each model was determined by performing both a threshold-dependent binomial test of omission and a threshold-independent receiver operating characteristic analysis. For the binomial test of omission, a threshold of 0.1 was selected from the output generated by Maxent; a *p*-value <0.05 was used to indicate whether the niche model outperformed a random model. For the threshold-independent receiver operating characteristic analysis, which produces a curve of sensitivity vs. 1-specificity, only models with an area under the curve (AUC) greater than 0.70 were retained.

A correlation matrix was generated for all environmental variables to test for collinearity. Variables with a correlation coefficient <0.6 and representing biologically important criteria for vector species were retained for model development. A model was constructed for each time period using land cover as a categorical variable and temperature, vegetation indices, elevation, and human population density as continuous variables. A step-wise selection procedure was used, and accuracy assessments were applied to evaluate the goodness-of-fit of each model. Maxent fits the model on features that are transformations of the covariates allowing potentially complex relationships to be modeled (39). The contribution of variables to the final model were assessed using two metrics: percent contribution, which is sensitive to path taken by the Maxent algorithm to reach the final model, and permutation importance, which is obtained by randomly permuting the values of each variable in turn and measuring the decrease in AUC, and thus more representative of covariate importance (38).

Environmental layers that were applied in the final models include (i) vegetation indices: NDVI_W_ (in 2014) and EVI_W_ (in 2004); (ii) temperature indices: *T*_H_ and *T*_C_; (iii) land cover; (iv) elevation; and (v) human population density. Ten model replicates were run for each time period, each with a random partitioning of training and test data. The raster maps of probability of suitability output by Maxent were averaged to determine the probability of suitability for each grid cell. The averaged probability of suitability map for each time period was converted into a binary map of predicted suitable and non-suitable areas using ArcGIS by applying the minimum predicted suitability of training records as the threshold value (40).

### Change in Vector Distribution and Environmental Factors

Binary model outputs were reprojected to Albers Equal Area spatial reference system in ArcGIS to compare the area of loss and gain in predicted vector habitat between years. Differences in environmental factors were calculated using the zonal statistics function in ArcGIS to assess changes over the 10-year period. Changes in the area of specific land cover classes were investigated using a land cover conversion matrix. To further compare model predictions of areas suitable for *An. arabiensis* between years, a forecasting model was developed by applying coefficients from the 2004 Maxent model to the 2014 environmental covariate layers; model replicates and threshold values were applied to develop a binary map as described above.

### Ethical Approval

This entomological study does not involve human subjects and was not subject to ethical approval. It is associated with a larger study protocol (NIMR/HQ/R.8a.Vol. IX/1898) that has been approved by the Tanzanian National Institute for Medical Research and the Ottawa Health Sciences Network Research Ethics Board (20150199-01H).

## Results

### Vector Niche Models

**Figure 2:**
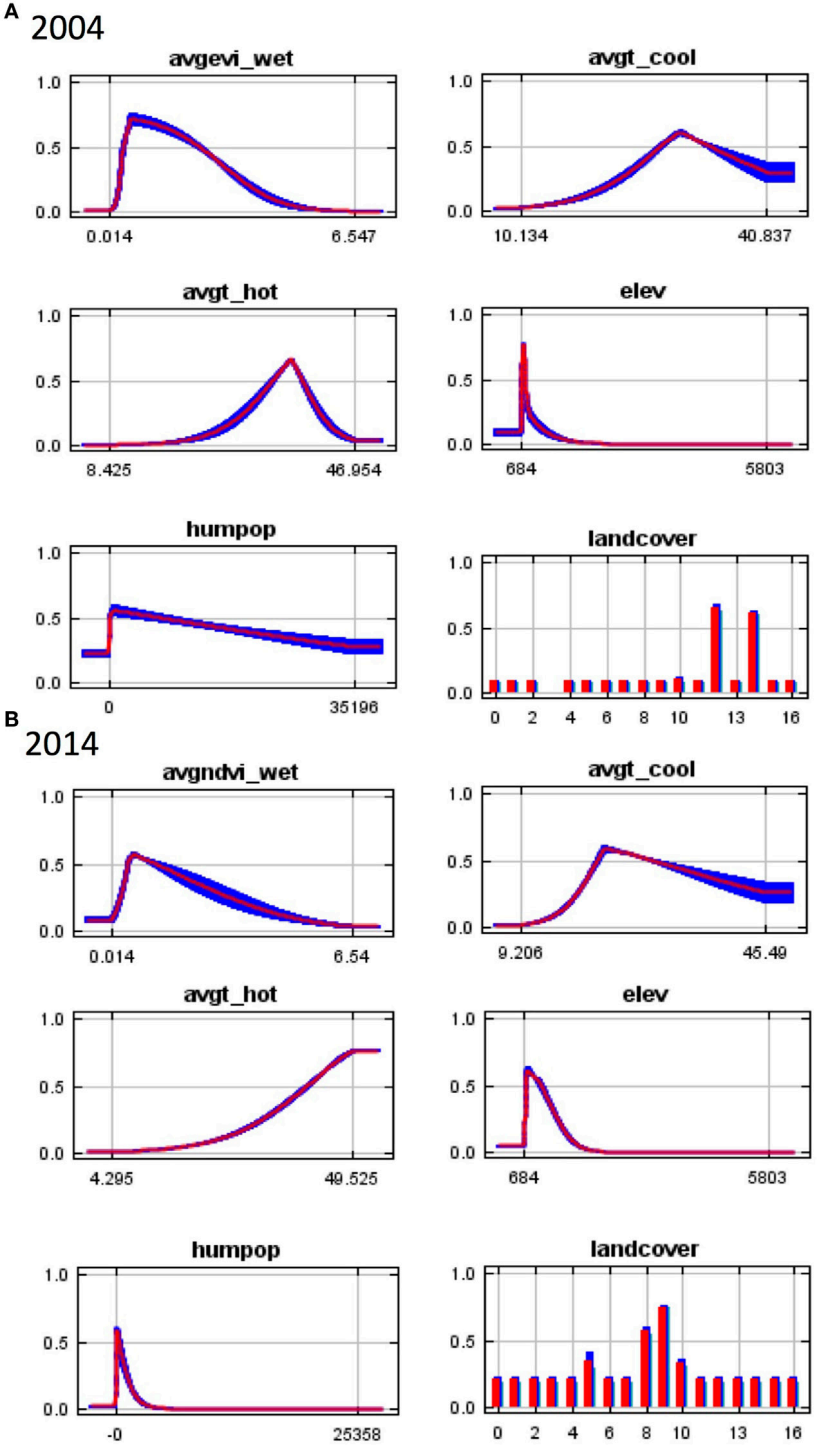
**Response curves for Maxent models in (A) 2004 and (B) 2014 showing the dependence of predicted suitability for *Anopheles arabiensis* occurrence on the selected variable accounting for dependencies induced by correlations with other variables**. Wet season NDVI (avgndvi_wet), wet season EVI (avgevi_wet), mean temperature of the warmest month (avgt_hot), mean temperature of the coldest month (avgt_cool), elevation (elev), human population density (humpop). NDVI, normalized differential vegetation index; EVI, enhanced vegetation index.

In 2004, elevation was the primary predictor of *An. arabiensis* distribution in the study area with a permutation importance of 52.3%, while wet season vegetation index was of secondary importance to the model (27.5%) (Table 1). Predicted suitability declined markedly with increasing altitude (Figure 2A), consistent with the exponential decline in vector density observed with increasing altitude in the Hai district during this time period (32). Land cover also contributed substantially to the model, while temperature and human population density were of relatively minor importance (Table 1).

**Table 1:** **Variable contributions to the Maxent model, 2004 and 2014**.

Year	Variable	Percentage contribution to model (%)	Permutation importance (%)	Model AUC
2004	Elevation	45.1	52.3	0.939
	Land cover	32.6	16.4	
	EVI_W_	15.2	27.5	
	*T*_H_	5.3	3.5	
	Human	1.7	0	
	Population			
	Density			
	*T*_C_	0.2	0.4	
2014	Human	51.1	24.2	0.907
	Population			
	Density			
	Land cover	25.9	16.8	
	Elevation	15.3	44.7	
	*T*_H_	5.6	11.3	
	NDVI_W_	2.1	2.9	
	*T*_C_	0	0.1	

*EVI, enhance vegetation index; NDVI, normalized differential vegetation index; T_H_, mean temperature of the warmest month; T_C_, mean temperature of the coldest month; AUC, area under the curve*.

In 2014, elevation contributed the most to the model with a permutation importance of 44.7%. Human population density was the second most important factor associated with *An. arabiensis* occurrence contributing 24.2%. Higher human population density was associated with decreasing *An. arabiensis* suitability (Figure 2B), consistent with the preference of *An. arabiensis* for rural environments. Land cover and mean temperature of the hottest month also contributed substantially to the model with wet season precipitation and mean temperature of the coolest month of relatively minor importance (Table 1).

Land cover was consistently one of the three most important factors in predictive models of *An. arabiensis* distribution in 2004 and 2014 (Table 1). Despite the similar contribution of land cover to both models (permutation importance of 16.4 and 16.8%, respectively), the types of land cover associated with *An. arabiensis* occurrence differed noticeably between 2004 and 2014 models. Cropland and cropland/natural vegetation mosaic (MODIS land cover classes 12 and 14, respectively) were positively associated with suitable habitat in 2004, while mixed forest, woody savannas, and savannas (MODIS land cover classes 5, 8, and 9, respectively) contributed the most to model predictions in 2014.

In both years, the response curves illustrate that increasing hot season temperature was associated with increasing suitability for *An. arabiensis* occurrence; however, the curves differed between models. In 2004, a maximum suitability for *An. arabiensis* occurrence was reached at approximately 35°C, with subsequent decline in predicted suitability with increasing temperature. In 2014, no maximum temperature was observed, possibly due to interaction with other variables. Predicted suitability for *An. arabiensis* initially increased with increasing wet season vegetation index but reached a peak after which suitability declined. This is consistent with the species’ preference for open sunlit aquatic habitats to support oviposition and larval development (34).

The projected model, in which the 2004 model algorithm was used to predict suitable habitat in 2014 based on 2014 environmental conditions, predicted less expansion in vector range in highland zones than the 2014 model; however, the contraction of species range at lower elevations was predicted consistently. The projected model only predicted 4/24 (16.7%) of the 2014 vector occurrence records, in contrast to the 2014 model that successfully predicted 23/24 (95.8%); this is possibly due to fewer samples collected at higher altitudes in 2004 but could also reflect changes in species habitat preference given the changes in land cover associations noted above. Thus, the 2014 model was retained to analyze the change in *An. arabiensis* range.

**Figure 3:**
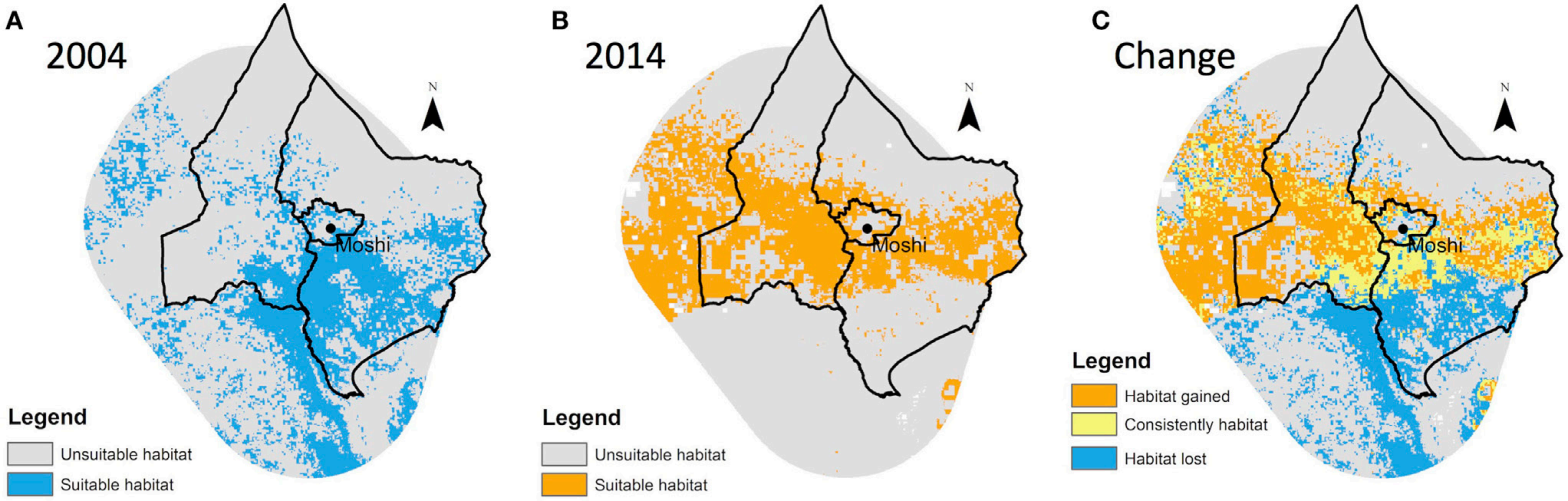
**Predicted distribution of *Anopheles arabiensis* in (A) 2004, (B) 2014, and (C) change showing areas of overlap, loss, and gain**.

The predicted area of suitable vector habitat in each year, as an estimate of the potential distribution of *An. arabiensis*, is shown in Figure 3.

### Change in Predicted Vector Distributions and Environmental Factors

Based on comparison of species niche models for 2004 and 2014, there was a substantial contraction of *An. arabiensis* range at lower altitudes over the 10-year period (Figure 3) with a net 325 km^2^ loss in predicted species range. In contrast, an expansion of *An. arabiensis* range was predicted at mid and high altitudes, with net gains of 219 and 194 km^2^, respectively.

**Figure 4:**
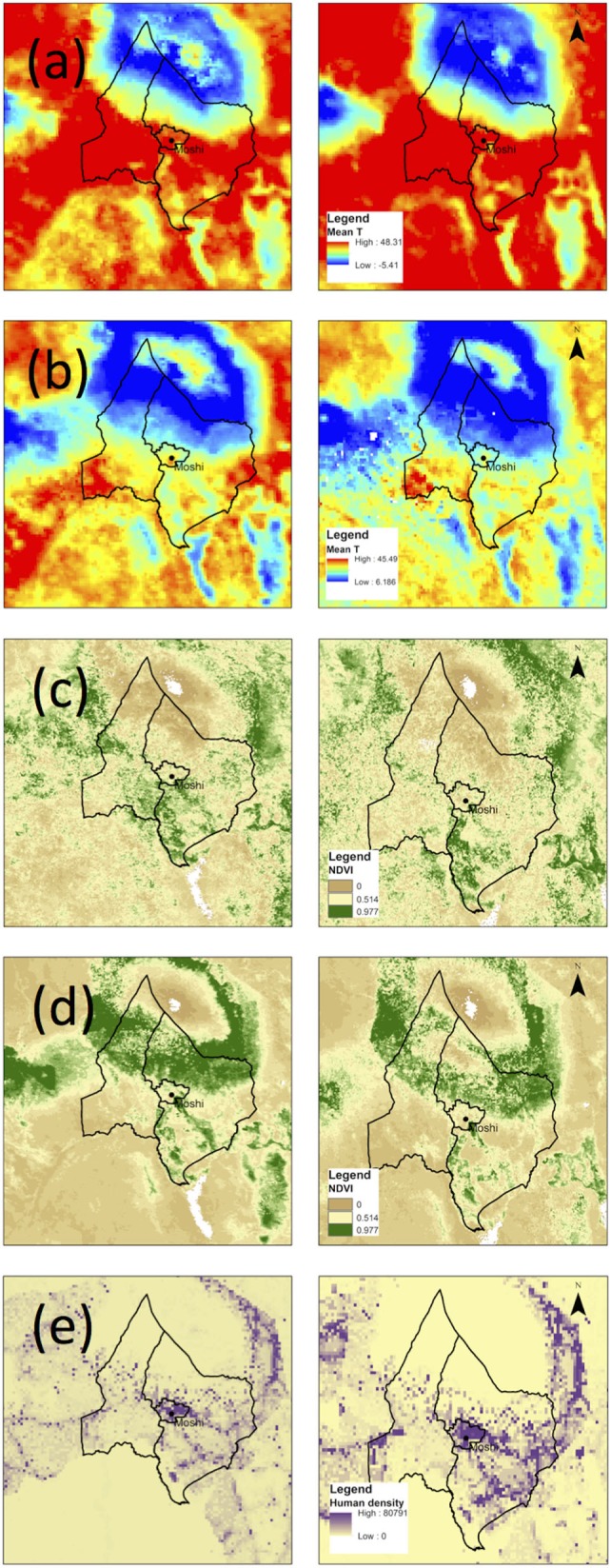
**Maps of environmental factors (A) *T*_H_, (B) *T*_C_, (C) NDVI_W_, (D) NDVI_D_, and (E) human population density in the study area in 2004 (left column) and 2014 (right column)**.

Changes in environmental factors across the study area are shown in Figure 4. From 2004 to 2014, an increase in the mean temperature of the warmest month was observed at all altitudes, alongside a decrease in the mean temperature of the coldest month (Table 2). Vegetation indices declined at low altitudes in the wet and dry seasons, indicating a trend toward lower precipitation. At mid and high altitudes, the dry season vegetation indices declined, while wet season indices increased, suggestive of increasing seasonal variability in precipitation. Human population density increased at all altitudes and was particularly pronounced at mid-altitude ranges where the major population centers are located: Boma Ng’ombe town in Hai district and Moshi town in Moshi district.

**Table 2:** **Change in climatic factors and human population density in the study area by altitude zone, 2004–2014**.

Altitude zone	Mean *T* of warmest month			Mean *T* of coldest month			Wet season NDVI			Dry season NDVI			Human population density		
	2004	2014	Net change	2004	2014	Net change	2004	2014	Net change	2004	2014	Net change	2004	2014	Net change
Low (≤900 m)	37.3	39.8	2.51	31.7	29.6	−2.1	0.59	0.58	−0.01	0.53	0.46	−0.07	261.3	282.82	21.5
Mid (901–1,000 m)	38.1	40.6	2.56	32.4	28.3	−4.2	0.42	0.46	0.04	0.32	0.29	−0.04	185.1	229.5	44.3
High (1,001–2,000 m)	32.9	34.5	1.62	27.5	24.4	−3.2	0.48	0.5	0.02	0.48	0.43	−0.05	124.5	147.11	22.6
Average	36.1	38.31	2.23	30.6	27.4	−3.1	0.5	0.52	0.02	0.45	0.39	−0.05	190.31	219.8	29.5

*NDVI, normalized differential vegetation index*.

A shift in land cover toward woody savannas in the highlands and grasslands at all altitudes was observed (Table 3). There was no discernable increase in land area classified as cropland at higher altitudes, but the loss of mixed and deciduous forest and concurrent increase in land covers with lower density of vegetation supports the occurrence of deforestation through natural and/or anthropogenic means.

**Table 3:** **Change in area of land cover (km^2^) in the study area by altitude zone, 2004–2014**.

Altitude zone	Croplands			Savannas and woody savannas			Mixed forest			Deciduous broadleaf forest			Grassland		
	2004	2014	Net change	2004	2014	Net change	2004	2014	Net change	2004	2014	Net change	2004	2014	Net change
Low (≤900 m)	649.1	306.0	−343.1	156.0	87.4	−68.6	5.36	4.3	−1.0	2.5	1.4	−1.2	662.7	1,028.4	365.7
Mid (901–1,000 m)	117.2	32.2	−85.1	46.1	55.0	8.9	10.2	4.7	−5.6	1.2	1.0	−0.1	285.9	390.6	104.7
High (1,001–2,000 m)	347.9	143.1	−204.8	367.8	594.0	226.2	96.2	48.4	−47.8	21.0	15.2	−5.8	569.1	648.2	79.1
Total	1,114.3	481.3	−633.0	569.9	736.4	166.5	111.8	57.4	−54.4	24.6	1,766	−7.1	1,517.6	2,067.2	549.5

## Discussion

The increasing availability and quality of satellite remote sensing data, combined with advances in species distribution modeling, has facilitated novel approaches to study changes in environmental conditions and disease vector species’ ranges over time. We show that climate, land use, and population changes have occurred that may have had an important influence on the distribution of *An. arabiensis*, the principal malaria vector species in a highland area of northern Tanzania. In particular, *An. arabiensis* has undergone a noticeable range expansion at higher altitudes with concurrent range contraction at lower altitudes. This has serious implications for the risk of malaria transmission among highland populations, which may be at heightened vulnerability due to their lack of previous malaria exposure (16).

By investigating the changes in environmental conditions and human population density that have taken place between 2004 and 2014 in two districts of Tanzania, we show that an average 2°C increase in warm season temperature has occurred alongside an average 30-person/km^2^ increase in human population density and substantial changes in land cover. These changes are associated with known impacts on malaria vector biology and ecology and would be expected to influence the range of *Anopheles* mosquitoes (41), in addition to the impacts of targeted vector control interventions in reducing vector populations (42).

Larval development of *An. gambiae* s.l. is highly temperature-dependent, requiring a minimum temperature of 16°C, while temperatures above 34°C have negative impacts on the survival of adult mosquitoes (43). Thus, increasing ambient temperature in highland areas has been found to enhance vector survival and reproduction, while further enhancing vectorial capacity through temperature-dependent effects on vector gonotrophic cycle and parasite sporogonic development (20), leading to an increase in altitudinal range (16). The observed increase in warm season temperature at all elevations, including highland areas at 1,000–2,000 m, is consistent with the observed altitudinal shift in *An. arabiensis* populations.

While a general drying trend was observed across the study area, as indicated by the change in wet and dry season vegetation indices, these effects were more pronounced at low elevations. The effects of precipitation on malaria vector ecology are well studied, and higher levels of precipitation have been shown to increase larval habitat availability (20, 44). Given the predicted range of *An. arabiensis* at higher elevations in 2004 and its persistence in 2014, this suggests that vector reproduction in highland areas may not be limited by precipitation to the same extent as lower elevations. Indeed, annual mean rainfall is typically much higher at higher elevations, with 800–900 mm common in lowland areas and >1,200 mm in highland areas (32).

Deforestation has been demonstrated to increase local temperatures and create microclimates conducive to sustaining populations of *Anopheles* mosquitoes. A study in western Kenya found that *An. arabiensis* is particularly well adapted to highland areas that have been deforested (20), which may explain the presence of this species at higher altitudes in recent years despite its reduction at lower altitudes. The observed deforestation on the slopes of Mount Kilimanjaro has several explanations, including the increasing demand for forest products and land for subsistence agriculture associated with population growth. Deforestation in Kilimanjaro has also been attributed to the impact of forest fires caused by traditional honey gathering activities (45) and climate changes (46). While agricultural expansion does not seem to be a major contributor to deforestation in our study, based on interpretation of the MODIS land cover data that shows minimal increase in land area classified as croplands, the increase in human population density has likely contributed to environmental modifications that support the creation of vector habitat.

A synergistic effect of temperature and precipitation has been described that may have important consequences for highland malaria epidemics (20), a phenomenon that has been observed in this region of Tanzania. Kulkarni et al. (32) found that densities of *An. arabiensis* in the Hai district peaked following the short rainy season that coincided with high ambient temperatures. Studies in the nearby Usambara Mountains of Tanzania found that low temperatures limited malaria transmission in the highlands during the cool season rains, and highland malaria transmission occurred during the warm dry season where it was maintained at very low vector densities (47). Given the trend of increasing precipitation variability predicted with future climate change (48), the occurrence of intense rainfall during periods of high ambient temperature may occur with greater frequency in future years, placing populations at greater risk of sporadic malaria epidemics.

While efforts were made to use comparable datasets to estimate the changes in vector range and environmental conditions that have occurred in the study area over a 10-year period, this study has several limitations. A smaller number of occurrence records were available from the highland and mid-altitude zones in 2001–2004 compared to low elevations, owing to the lower number of sampling locations as well as lower rates of species detection at higher altitudes. To mitigate potential sampling bias, entomological collections in 2014–2015 were conducted over two consecutive nights in each site during the period following the short and long rainy seasons when *Anopheles* mosquitoes are most abundant and followed a similar approach that was used in entomological collections a decade earlier; furthermore, sampling effort was consistent in all altitude zones. In 2014–2015, outdoor collections were further conducted to sample the exophagic population, recognizing that *An. arabiensis* behavior may differ according to ambient temperature, with higher endophily noted in cooler conditions (32). Thus, while the predicted degree of expansion of vector range at higher altitudes should be interpreted cautiously given the differences in sampling that may have influenced model results, the predicted contraction of vector range at lower altitudes is most likely real and consistent with climate and land cover changes observed during the 10-year period, mirroring declines in malaria prevalence among local populations.

It is important to note that environmental changes alone may not explain the observed trends in vector populations and malaria prevalence, since scale-up of malaria control interventions has occurred simultaneously. Free distribution of long-lasting insecticidal nets (LLINs) began in Tanzania in late 2008 resulting in a rapid increase in coverage, while prior to this the commercial sector comprised the main distribution channel of ITNs, alongside subsidized provision through government health programs (26). Changes in antimalarial drug policy also occurred, with first-line treatment switching from chloroquine to sulphadoxine/pyrimethamine in 2001 and subsequently to the artemisinin combination therapy artermether–lumefantrine in 2006 (49).

The role of vector control interventions could not be directly evaluated in the models due to data limitations; available data on LLIN coverage reflect only a limited geographic sample from prior targeted studies, which are conducted at different time points, or from national surveys designed to estimate district- or regional-level LLIN coverage indices (50), which biases finer-scale analyses. Nonetheless, previous studies in this region of northern Tanzania have found similar rates of bednet ownership in villages situated at low (750 m) and mid-altitudes (1,050 m) (51), therefore, the impact of interventions would likely be similar across the study area. It is possible that the scale-up of vector control interventions has acted synergistically with climate to contribute to the observed declines of vector populations at lower elevations; however, further predictions incorporating multiple time points over the 10-year period would be needed to discern these effects. Interestingly, the declines in malaria transmission and malaria vector densities observed over a 10- to 20-year period in Muheza district of northeastern Tanzania were attributed not only to increased use of ITNs but also to changes in climate, improvement in socioeconomic status, and human land use activities, although these factors were not measured directly (21, 52).

Despite the reduction in malaria vector populations and malaria transmission at lower elevations, the persistence of malaria vectors at higher altitudes, where their range coincides with the areas of highest human population density, may present a future public health concern in this region of Tanzania. Given that environmental conditions in the mid-altitude and highland zones are conducive to *An. arabiensis* population growth and epidemic malaria transmission, and populations are biologically vulnerable to malaria, there could be an increased risk of malaria introduction if transmission in the lowland areas increases. A resurgence in the lowlands could occur if control measures are not sustained or fail as a result of drug and insecticide resistance, or if favorable climatic conditions return. While the Hai and Moshi districts of Tanzania have experienced low prevalence and incidence of malaria in the last decade, a heightened suitability for *An. arabiensis* vectors (41) and risk of malaria transmission (15) is predicted to occur in this region of East Africa with future climate change. Recognizing that the impacts of environmental changes and interventions on malaria transmission are complex, it has been suggested that integrative approaches are needed that address individual vulnerabilities to the disease, including socioeconomic, behavioral, environmental, and political aspects in addition to malaria prevention and control, particularly as countries achieve very low levels of transmission (7). Ongoing spatial modeling of vector species and identification of malaria transmission hotspots in these low transmission areas may help to inform the optimal targeting and timing of interventions, in order to prevent disease emergence and reduce health impacts in highland communities.

## Author Contributions

MK conceived the study and designed the experiments. RD contributed to study design and assembled the spatial data. EK, MK, RK, DK, NP, and FM collected the entomological data and performed analyses. RD, AT, and MK analyzed and interpreted the data. MK wrote the paper, and all the authors contributed to interpreting the data and critically revised the manuscript. This study was funded by Grand Challenges Canada grant no. S6-0476-01-10 to RD, MK and FM and a University of Ottawa Faculty of Medicine operating grant to MK.

## Conflict of Interest Statement

The authors declare that the research was conducted in the absence of any commercial or financial relationships that could be construed as a potential conflict of interest.
